# Variations in signal‐to‐noise characteristics of tissue‐equivalent attenuators for mammographic automatic exposure control system performance evaluation

**DOI:** 10.1002/acm2.13870

**Published:** 2022-12-15

**Authors:** Courtney K. Morrison, Erin B. Macdonald, Nicholas B. Bevins

**Affiliations:** ^1^ Department of Radiology Henry Ford Health Detroit Michigan USA; ^2^ Department of Radiology Duke University Medical Center Durham North Carolina USA

**Keywords:** automatic exposure control, breast‐equivalent attenuators, mammography, quality control

## Abstract

**Purpose:**

This work investigates the impact of tissue‐equivalent attenuator choice on measured signal‐to‐noise ratio (SNR) for automatic exposure control (AEC) performance evaluation in digital mammography. It also investigates how the SNR changes for each material when used to evaluate AEC performance across different mammography systems.

**Methods:**

AEC performance was evaluated for four mammography systems using seven attenuator sets at two thicknesses (4 and 8 cm). All systems were evaluated in 2D imaging mode, and one system was evaluated in digital breast tomosynthesis (DBT) mode. The methodology followed the 2018 ACR digital mammography quality control (DMQC) manual. Each system‐attenuator‐thickness combination was evaluated using For Processing images in ImageJ with standard ROI size and location. The closest annual physicist testing results were used to explore the impact of varying measured AEC performance on image quality.

**Results:**

The measured SNR varied by 44%–54% within each system across all attenuators at 4 cm thickness in 2D mode. The variation appeared to be largely due to changes in measured noise, with variations of 46%–67% within each system across all attenuators at 4 cm thickness in 2D mode. Two systems had failing SNR levels for two of the materials using the minimum SNR criterion specified in the ACR DMQC manual. Similar trends were seen in DBT mode and at 8 cm thickness. Within each material, there was 115%–131% variation at 4 cm and 82%–114% variation at 8 cm in the measured SNR across the four imaging systems. Variation in SNR did not correlate with system operating level based on visual image quality and average glandular dose (AGD).

**Conclusion:**

Choice of tissue‐equivalent attenuator for AEC performance evaluation affects measured SNR values. Depending on the material, the difference may be enough to result in failure following the longitudinal and absolute thresholds specified in the ACR DMQC manual.

## 1 INTRODUCTION

Automatic exposure control (AEC) systems in digital mammography control the technique parameters (e.g., target, filter, potential, current, time) of a mammographic acquisition to produce a similar detector signal for a given breast thickness and attenuation characteristics. The specific methodologies of how AEC systems accomplish this vary between vendors, but the goals remain largely the same. As a result of their importance in maintaining reliable image quality at an appropriate radiation dose, AEC systems are a vital component of any mammography system and should be included in any quality control (QC) program.

In 2016, the American College of Radiology (ACR) released the first edition of the digital mammography quality control (DMQC) Manual.^1^ This was the first QC manual created by the ACR since its 1999 edition, which was published in the screen‐film era. With the release of the ACR DMQC manual in 2016, facilities now have the option to follow the ACR DMQC manual as an alternative standard in lieu of manufacturers’ QC manuals.^2^ The ACR released the second edition of the ACR DMQC manual in 2018 to include systems that use digital breast tomosynthesis (DBT).

The ACR DMQC manual uses a generalized method for performance evaluation of AEC systems, likely due to the wide variety of vendor implementations of AEC methodologies. As part of a mammography equipment evaluation (MEE) (following either installation or relevant service) and annually, the physicist is required to perform an evaluation of the AEC system across a variety of compression thicknesses and available imaging modes. Physicists must use “tissue‐equivalent attenuators” of approximate thicknesses to match each required compression thickness. The majority of the performance criteria for the AEC evaluation stress longitudinal consistency: the measured signal‐to‐noise (SNR) must be within ±15% of the last MEE's SNR for each thickness and imaging mode. There is one absolute pass/fail criterion: the SNR with 4 cm of tissue‐equivalent attenuator in 2D mode must be greater than or equal to 40.

The 2018 ACR DMQC manual provides a list of example materials, including poly(methyl methacrylate) (PMMA), BR‐12,^3^ and BR‐50. Additionally, the manual provides a caveat that the tissue‐equivalent attenuators do not have the same attenuation characteristics as breast tissue. However, the ACR DMQC does not provide guidance on, or precautions about, the appropriate selection of tissue‐equivalent attenuator for assessing AEC performance in the context of the new performance criteria. In this paper, we investigate how the composition of the tissue‐equivalent attenuator affects the measured AEC system performance and its impact on testing results.

## 2 METHODS

In this work, we evaluated the AEC system performance on four mammography models from two manufacturers: GE Senographe Essential with Senoclaire (GE Healthcare, Waukesha, WI), GE Senographe Pristina, Hologic Lorad Selenia (Hologic, Marlborough, MA), and Hologic Selenia 3Dimensions. The AEC system was evaluated in 2D mode for all four systems, and one unit was evaluated in DBT mode. Seven sets of tissue‐equivalent attenuators from four manufacturers (Gammex/Sun Nuclear, Middleton, WI; CIRS, Norfolk, VA; GE Healthcare, Waukesha, WI; and Unknown) were included in this work (Table 1), including four sets of BR‐12, two sets of PMMA, and one set of BR‐50 (1454 HE Breast 50/50). Two sets of BR‐12 were identical in that they were from the same manufacturer but different lots. One set of PMMA (PMMA 2) is sold in “nominally” 2 cm thickness slabs, with actual thickness of 0.75 inches (1.905 cm).

**TABLE 1:** Attenuating materials and manufacturers

Material	Description	Manufacturer
BR‐12	BR‐12 1	A
BR‐12	BR‐12 2	A
BR‐12	BR‐12 3	B
BR‐12	BR‐12 4	C
BR‐50	BR‐50	C
PMMA	PMMA 1	D
PMMA	PMMA 2	C

The AEC system performance was evaluated with 4 and 8 cm of attenuator in 2D mode, while DBT mode was only evaluated with 4 cm of attenuator. Only one of the identical BR‐12 sets was imaged at the 8 cm thickness. All measurements were made on the same day. While 2 and 6 cm of attenuator are also used to evaluate AEC system performance in the ACR DMQC manual, they were not included in this study.

**FIGURE 1:**
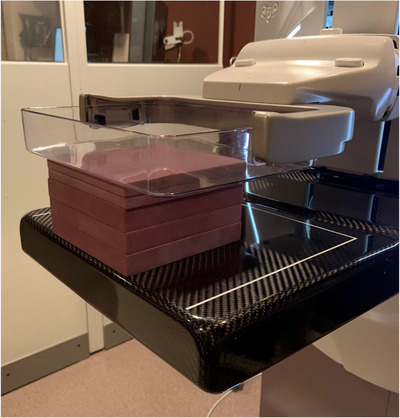
Experimental setup. The attenuating material was centered left‐to‐right and aligned to the chest‐wall edge of the image receptor.

The AEC system performance was assessed following the method described in the ACR DMQC manual. Briefly, the tissue‐equivalent attenuator was aligned with the chest‐wall edge of the breast support and centered laterally in the left‐right direction, as shown in Figure 1. The small compression paddle was used to compress to the actual thickness of attenuator. When compressing to the actual thickness was not possible, the paddle was compressed to 12 lbs or 5 daN, as specified in the ACR DMQC manual. An image was acquired in 2D mode using the large focal spot with an AEC mode that automatically selects the target/filter combination, tube potential, tube current, and exposure time. For GE systems, an LCC view was acquired in CNT or STD AOP mode, depending on the mode used clinically. For Hologic systems, a Flat Field view was acquired in Auto Filter AEC mode using photocell 2 and 0 density. The second photocell is 3 cm from the chest‐wall edge of the phantom and was used to ensure that the AEC sensor was covered by the attenuating material.

All image analysis was performed in ImageJ.^4^ A 1 cm^2^ rectangular ROI was automatically placed on each For Processing image with its center 3 cm from the chest‐wall edge of the image and centered laterally in the left‐right direction. For the DBT images, the ROI was placed on the central reconstructed plane. The mean signal value and standard deviation were recorded, and the SNR was calculated as the ratio of the mean signal to the standard deviation. For Hologic systems, the DC offset (50) was subtracted from the mean signal value before calculating the SNR. The relative percent differences between the mean signal values, standard deviations, and SNRs were used for comparison.

For additional comparison across the four systems, the annual physicist testing results were collected to review the ACR digital mammography (DM) phantom testing results (phantom score, contrast‐to‐noise ratio (CNR), SNR, and average glandular dose [AGD]). All annual testing was performed by one of the authors following the ACR DMQC manual guidelines, using the clinical imaging mode to acquire the phantom image. For these imaging systems, the clinical imaging mode is the same AEC mode as specified above for the AEC system performance evaluation.

## 3 RESULTS

For the AEC settings used, the use of different attenuator materials did not change the anode target material or filter material for each system. For Systems 1 and 2, the tube potential also remained constant across all attenuators at a given thickness. On Systems 3 and 4, the AEC systems used slightly lower techniques for PMMA 2, which is slightly thinner than the other attenuators. For System 3, PMMA 2 used 27 kV at 4 cm and 30 kV at 8 cm, compared with 28 kV at 4 cm and 32 kV for the other six attenuators. For System 4, PMMA 2 used 26 kV at 4 cm and 32 kV at 8 cm, compared with 27 kV at 4 cm and 33 kV at 8 cm for the other six attenuators. The tube potential and tube current‐time product (mAs) values for each mammography system and attenuator are summarized in Table 2.

**TABLE 2:** AEC‐selected technique parameters for exposures

	System 1		System 2		System 3		System 4	
	4 cm	8 cm	4 cm	8 cm	4 cm	8 cm	4 cm	8 cm
BR‐12 1	27/82	*NA*	34/26	*NA*	28/101	*NA*	27/71	*NA*
BR‐12 2	27/81	31/126	34/26	34/103	28/99	32/286	27/70	33/134
BR‐12 3	27/85	31/131	34/27	34/103	28/101	32/302	27/73	33/143
BR‐12 4	27/82	31/131	34/27	34/103	28/98	32/283	27/70	33/134
BR‐50	27/121	31/144	34/29	34/107	28/109	32/323	27/79	33/152
PMMA 1	27/116	31/184	34/33	34/109	28/116	32/399	27/86	33/191
PMMA 2	27/87	31/104	34/23	34/102	27/98	30/329	26/72	32/140

*Note*: Entries are formatted as tube potential (kV)/tube current‐time product (mAs).

**FIGURE 2:**
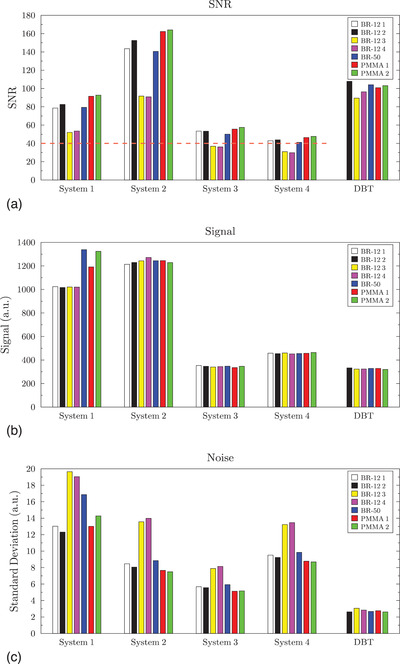
The SNR (a), mean background signal (b), and noise (c) results with 4 cm of tissue‐equivalent attenuator for the four mammography system models in 2D mode are shown, with DBT mode results from one system on the far right. The bars represent the different tissue‐equivalent attenuators.

A graph with the SNR results with 4 cm of attenuator is shown in Figure 2a. The white and black bars represent the two identical sets of BR‐12, which yielded high reproducibility with similar signal, noise, and SNR values (average differences of 1.1%, 3.9%, and 3.3%, respectively). The SNR across all attenuators in 2D mode exhibited large differences, ranging from 44% to 54%, with an average difference of 49%. In DBT mode, the difference was 18%.

The ACR absolute pass/fail criterion is an SNR greater than or equal to 40 in 2D mode, indicated by the dashed red line in Figure 2a. Four cases had an SNR less than 40 and thus would not meet the ACR criterion. Additionally, 46% would not meet the longitudinal criterion that the SNR be within ±15% of the MEE SNR if different attenuators were used at MEE and the annual test.

Looking at the signal and noise components separately (Figure 2b,c), the mean background signal in 2D mode did not vary when different attenuators were used (average difference of 10%); however, the noise exhibited variations similar to the SNR, with an average difference of 52%. One mammography system model in particular had unusually high variation in signal. Excluding this model, the difference in signal ranged from 3%–5%, while the difference in noise remained high, ranging from 46%–67%. In DBT mode, the difference in signal was 4%, and the difference in noise was 16%.

Comparing each material across all four mammography systems with 4 cm of attenuator, the SNR varied by 115%–131%, with an average variation of 125%. One system, in particular, System 2, showed much higher SNR than the others, despite operating at a level with a similar AGD.

**FIGURE 3:**
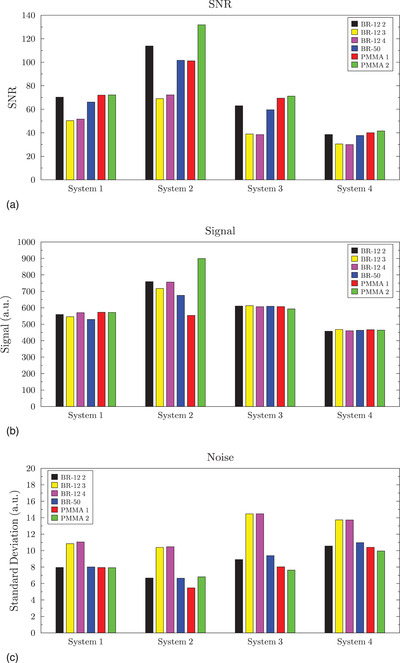
The SNR (a), mean background signal (b), and noise (c) results with 8 cm of tissue‐equivalent attenuator for the four mammography models in 2D mode are shown. The bars represent the different tissue‐equivalent attenuators.

Similar trends were observed with 8 cm of attenuator (Figure 3). The average differences in SNR, signal, and noise were 47, 15, and 49%, respectively. Again, one mammography system model had unusually high variations in signal. If we exclude this model, the signal difference ranged from 2%–8%, and the noise difference ranged from 36%–65%. If different attenuators were used at MEE and the annual test, 60% of these cases would not meet the ACR longitudinal pass/fail criterion. Across the mammography systems, the same material showed a variation of 82% to 114% in measured SNR.

Table 3 provides a summary of the ACR DM phantom results. The image quality assessment using the DM phantom scoring (fibers, speck groups, and masses) showed good agreement between all four systems, with all scores within 0.5 for a given object for the same DM phantom. Like the AEC test results with both 4 and 8 cm of attenuator, the SNR and CNR results for the DM phantom varied greatly by system. The AGD were all below the limit of 3 mGy, though there was variability ranging from 1.24 to 1.97 mGy. The AGD did not correlate with measured SNR or CNR.

**TABLE 3:** ACR DM phantom test results for each mammography system

	Phantom score					
Equipment	Fibers	Speck groups	Masses	SNR	CNR	AGD (mGy)
System 1	4.5	5.0	4.0	82.3	5.0	1.97
System 2	4.0	5.0	4.5	140.6	7.8	1.62
System 3	4.0	5.0	4.0	52.4	3.1	1.24
System 4	4.5	5.0	4.0	46.4	3.2	1.84

## 4 DISCUSSION

Two key observations can be made from the data presented in this work: first, the choice of tissue‐equivalent attenuator has a strong influence on the resulting SNR measurements; and second, the measured SNR varies greatly across different mammography systems, even for the same tissue‐equivalent attenuator, without corresponding variability in perceived image quality or system performance.

On the first point, the variety of allowable tissue‐equivalent attenuators in the ACR DMQC manual without guidance on the proper use or selection may lead to erroneous results when evaluating AEC system performance, for both longitudinal and absolute pass/fail criteria. In the case of Systems 3 and 4, BR‐12 3 and 4 both resulted in an apparent SNR failure with 4 cm of attenuator, while other approved materials easily passed, including other material also marketed as BR‐12. This is likely due to the fact that the tissue‐equivalent attenuators included in this work have different formulations, even those with the same name. Both BR‐12 and BR‐50 contain high‐atomic number particulate fillers to attain appropriate attenuation properties. However, the original formulation of BR‐12 contains hollow phenolic microsphere (PMS) powder,^3^ while the BR‐50 material used in this study contains glass bubbles to reduce the density.^5^ Among materials marketed as BR‐12, different manufacturers use different formulations, with some using the original BR‐12 defined as 50% fat/50% water equivalence^3^ and others using 47% glandular/53% adipose.^6^ In contrast to the “BR” materials, which contain added fillers that control the attenuation properties and density of the materials, PMMA is inherently non‐structural and contains no added fillers. These compositional differences may cause differences in the structural noise characteristics of these materials and likely contribute to the variations in noise that were observed in this work. The technique parameters shown in Table 2 suggest the noise differences are not due to changes in AEC‐selected technique. All four BR‐12 attenuators used nearly identical techniques within each system, despite large variations in noise characteristics. Furthermore, different attenuators (e.g., BR‐12 1 and 2 vs. PMMA 1) used different techniques, but resulted in similar SNR, signal, and noise measurements.

For the second observation, the results show that across different mammography system manufacturers and models, the measured SNR (and CNR for the DM phantom) may vary by nearly a factor of three for a given attenuator. This increase in SNR does not correspond with any apparent change in image quality, as all four systems demonstrated similar visual image quality using the same ACR DM phantom. In addition, the measured SNR does not simply scale with the AGD between systems. For example, System 4 consistently demonstrated the lowest SNR, as shown in Figures 2 and 3, and Table 3, yet had one the highest measured AGDs. This is likely due to the variation in target/filter combinations, detector design (both material and pixel pitch), grid specifications, and image processing that occurs on both the “For Processing” and “For Presentation” images generated by each system. Since SNR is highly dependent on the entire imaging chain, particularly the detector, it is likely not well suited for comparing system performance across different manufacturers and models.

This study only evaluated the AEC system performance with different tissue‐equivalent attenuators on mammography systems from two manufacturers. In order to better understand the impact of tissue‐equivalent attenuator choice on the AEC system performance evaluation, further studies could perform similar measurements on additional manufacturers' systems. In addition, more samples of tissue‐equivalent attenuators could be used to further investigate the impact of material choice on measured system performance.

This work examines the effect of different tissue‐equivalent attenuators on the AEC system performance largely within the framework of the methodology specified in the ACR DMQC manual. Other AEC system evaluation approaches, such as those specified within vendor‐specific QC manuals or the IEC standards,^7^, ^8^ use alternative methodology and/or standardized attenuator materials (e.g., only PMMA), which likely reduce the variability and potential for erroneous failures seen in this work.

## 5 CONCLUSION

The choice of tissue‐equivalent attenuator can impact the measured AEC system performance, particularly when the evaluation is based on longitudinal changes in SNR or absolute SNR values. Differences in SNR as high as 58% were observed in this study, without a concordant change in apparent imaging system performance. These differences are likely due to the structural noise characteristics of the attenuators, as demonstrated by the low signal differences but high noise differences. Care should be taken in attenuator selection as the measured AEC system performance may not reflect the true system performance.

In addition, care should be used when establishing and using absolute pass/fail criteria for SNR and/or CNR across different manufacturers and models of systems. Passing thresholds which may be appropriate for one manufacturer may not be appropriate for another, even for similar system performance and attenuating material. The use of such values may result in erroneous results on one system, while simultaneously missing needed action on another.

## AUTHOR CONTRIBUTION

All authors contributed to the study design, collection of data, analysis of results, and preparation of this manuscript.

## CONFLICT OF INTEREST

No conflicts of interest.
